# Inhibitory effects of Ponciri Fructus on testosterone-induced benign prostatic hyperplasia in rats

**DOI:** 10.1186/s12906-017-1877-y

**Published:** 2017-08-03

**Authors:** Woo-Young Jeon, Ohn Soon Kim, Chang-Seob Seo, Seong Eun Jin, Jung-Ae Kim, Hyeun-Kyoo Shin, Yong-ung Kim, Mee-Young Lee

**Affiliations:** 1 K-herb Research Center, Korea Institute of Oriental Medicine, 1672 Yuseong-daero, Yuseong-gu, Daejeon, 34054 Republic of Korea; 2 KM Convergence Research Division, Korea Institute of Oriental Medicine, 1672 Yuseong-daero, Yuseong-gu, Daejeon, 34054 Republic of Korea; 3 School of Pharmacy, College of Pharmacy, Yeungnam University, 280 Daehak-ro, Gyeongsan-si, Gyeongsangbuk-do 38541 Republic of Korea; 4 Department of Pharmaceutical Engineering, College of Biomedical Science, Daegu Haany University, 1 Hanuidae-ro, Gyeongsan-si, Gyeongsangbuk-do 38610 Republic of Korea

**Keywords:** Ponciri Fructus, Benign prostatic hyperplasia, Dihydrotestosterone, Antioxidant enzymes, Proliferating cell nuclear antigen

## Abstract

**Background:**

Benign prostatic hyperplasia (BPH) is non-cancerous condition of enlargement of the prostate, a common occurrence in older men. The immature fruits of *Poncirus trifoliata* (L.) Rafinesque (Rutaceae), Ponciri Fructus are widely used in traditional oriental medicine for the therapy of various diseases. However, little is known about the mechanism underlying the pathogenesis of BPH. In the present study, we investigated the protective effects of a Ponciri Fructus extract (PFE) on the development of BPH in a in a rat model of BPH induced by testosterone propionate (TP).

**Methods:**

Male Sprague Dawley rats were used as a model of BPH after its induction by daily subcutaneous injections of TP/corn oil, for a period of four weeks. PFE was administrated daily 1 h before TP/corn oil injection by oral gavage at a dose level of 200 mg/kg during the 4 weeks of TP/corn oil injections. All rats were sacrificed at the end of the experiment, we measured the relative prostate weight, the levels of testosterone and dihydrotestosterone (DHT), histological changes, activities of antioxidant enzymes (catalase, glutathione peroxidase, glutathione reductase, and superoxide dismutase), and expression of proliferating cell nuclear antigen (PCNA). In addition, we also measured the inhibition (%) of 5α-reductase in the prostatic tissue.

**Results:**

Our findings indicate that PFE significantly inhibited the development of BPH; decreased the relative prostate weight, the level of testosterone and DHT in serum and prostatic tissue, prostatic hyperplasia, expression of PCNA, and increased the antioxidant enzymes. Moreover, PFE showed a weak inhibitory activity on 5α-reductase.

**Conclusions:**

These results suggest that PFE may be used as a therapeutic agent for BPH via antiproliferative and antioxidant effects.

## Background

Benign prostatic hyperplasia (BPH) is one of the most common diseases of aging in men and is characterized by progressive hyperplasia of glandular and stromal tissues, leading to an enlarged prostate size [1]. An enlarged prostate can constrict the urethra, leading to severe urinary problems including a weak urinary stream, dysuria, urinary frequency, bladder outlet obstruction, and incomplete bladder emptying [2]. Biochemically, an imbalance of androgen/estrogen and overexpression of growth factors plays an important part in the development and progression of BPH [3]. Dihydrotestosterone (DHT), a metabolite of testosterone and a crucial mediator of prostate growth, is synthesized in the prostate from circulating testosterone by 5α-reductase and is a highly lipophilic enzyme found on intracellular membranes [4]. 5α-reductase exists in two isoforms: Type 1 and Type 2. Finasteride, a synthetic 5α-reductase Type 2 inhibitor, is used to treat BPH [5].

Oxidative stress plays a critical role in prostatic hyperplasia and is responsible for an imbalance between the production of free radicals and scavenging of free radicals, and can injure important components of tissues such as mRNA, DNA, and proteins [6]. Many studies have investigated antioxidants in the prevention and treatment of BPH [7, 8], and antioxidants have been proposed as therapeutic agents to prevent the progress of BPH by increasing the activities of antioxidant enzymes such as catalase (CAT), glutathione peroxidase (GPx), glutathione reductase (GR), and superoxide dismutase (SOD).

The immature fruits of *Poncirus trifoliata* (L.) Rafinesque (Rutaceae), Ponciri Fructus are traditionally used for the treatment of common edema, dyspepsia, and constipation in many Asian countries [9]. Many pharmacological studies have shown that Ponciri Fructus exerts anti-inflammatory [10] and gastroprotective effects [11] in vitro and in vivo. Ponciri Fructus contains high amounts of various compounds, including flavonoids and coumarins [11]. They demonstrate an extensive range of biological activities including antiallergic, antitumor, antiviral, and cancer chemopreventive properties. In the present study, in vivo experiments were performed to confirm the effect of Ponciri Fructus extract (PFE) in an animal model of BPH, because its effect has proved in vitro using a myofibroblast stromal cell line-derived from normal adult prostate (WPMY-1) in our laboratory (data not shown). Although many studies have investigated the pharmacological effects of PFE, there is little information regarding its effects on the mechanism underlying the pathogenesis of BPH. Therefore, we aimed to investigate the antiproliferative and antioxidant effects of PFE related to oxidative stress on the development of BPH in a rat model of BPH induced by testosterone propionate (TP).

## Methods

### Plant materials

Dried fruit of *P. trifoliata* were purchased from Kwangmyungdang (Ulsan, Korea) in November 2014 and taxonomically confirmed by Dr. Jung Hoon Kim at the Division of Pharmacology, School of Korean Medicine, Pusan National University (Yangsan, Gyeongnam 626–870, Korea). A voucher specimen (2014-GO-10) has been deposited at the K-herb Research Center, Korea Institute of Oriental Medicine (KIOM).

### Chemicals and reagents

Neoponcirin, poncirin, and auraptene were purchased from ChemFaces (Wuhan, China). Umbelliferone, naringin, and imperatorin were obtained from Shanghai Sunny Biotech (Shanghai China), Sigma-Aldrich (St Louis, MO, USA), and ChromaDex (Irvine, CA, USA), respectively. The purity of the six reference standards was ≥95.0%. HPLC-grade methanol, acetonitrile, and water were obtained from J.T. Baker (Phillipsburg, NJ, USA). Glacial acetic acid, analytical reagent grade was purchased from Merck (Darmstadt, Germany). Other reagents were as follows: TP (T0028; Tokyo Chemical Ins. Co., Tokyo, Japan), corn oil (C8267; Sigma-Aldrich), (−)-riboflavin (Rib) (R9504; Sigma-Aldrich), Finasteride (Fin) (F1293; Sigma-Aldrich), testosterone enzyme-linked immunosorbent assay (ELISA) kit (582,701; Cayman Chemical, Ann Arbor, MI, USA), DHT ELISA kit (11-DHTHU-E01; ALPCO Diagnostics, Salem, NH, USA), catalase assay kit (707,002; Cayman Chemical), GPx assay kit (703,102; Cayman Chemical), GR assay kit (703,202; Cayman Chemical), SOD assay kit (706,002, Cayman Chemical), anti-proliferating cell nuclear antigen (PCNA) antibody (ab-29; Abcam, Cambridge, MA, USA) and anti-β-actin antibody (4967S; Cell Signaling Technology, Danvers, MA, USA).

### Preparation of 70% ethanol extract of Ponciri fructus

Dried fruit of *P. trifoliata* (100.0 kg) were extracted with 70% ethanol (1000 L × 3 times) for 60 min at 80 °C using and electric extractor (Cosmos-660; Kyungseo Machine Co., Incheon, Korea). The extracted solution was filtered through filter paper, evaporated to dryness at 40 °C under vacuum (Eyela N-21NS, Tokyo, Japan), and freeze-dried (PVTFD10RS, IlShinBioBase, Yangju, Korea). The amount of 70% ethanolic extract obtained was 19.4 kg (19.4%).

### High-performance liquid chromatography (HPLC) analysis

The chromatographic analysis for simultaneous quantification of the three flavonoids (naringin, neoponcirin, and poncirin) and the three coumarins (umbelliferone, imperatorin, and auraptene) was performed using a Prominence LC-20A series (Shimadzu Co., Kyoto, Japan) consisting of a solvent delivery unit (LC-20AT), online degasser (DGU-20A_3_), column oven (CTO-20A), auto sample injector (SIL-20 AC), and photodiode array detector (PDA, SPD-M20A) as described previously [12]. The data acquired were processed using Lab Solution software (version 5.54 SP3, Shimadzu, Kyoto, Japan). Six compounds were separated on a SunFire C18 column (250 mm × 4.6 mm, 5 μm, Waters, Milford, MA, USA) maintained at 40 °C. The mobile phases consisted of 1.0% (*v*/v) acetic acid in distilled water (A) and 1.0% (*v*/v) acetic acid in acetonitrile (B). The gradient flow was as follows: 10%–65% B for 0–30 min, 65%–90% B for 30–35 min, 90% B for 35–40 min, and 90%–10% B for 40–45 min. The flow-rate was 1.0 mL/min and the injection volume was 10 μL. For quantitative determination, 100 mg of lyophilized sample was dissolved in 20 mL of distilled 70% methanol and then the solution was filtered through a 0.2 μm membrane filter (PALL Life Sciences, Ann Arbor, MI, USA) before HPLC.

### Animals

Male 6-week-old Sprague Dawley (SD) rats weighing 250–350 g (Orientbio Inc., Seoul, Korea) were housed in a room maintained at 18–23 °C with a relative humidity of 40–60%, and an alternating 12 h light/12 h dark cycle. Rats were provided with a standard laboratory diet and water ad libitum. All experimental procedures were conducted in accordance with the NIH Guidelines for the Care and Use of Laboratory Animals and were approved by the Institutional Animal Care and Use Committee of the Chungnam National University (animal ethics approval number: CNU-00446). Animal handling followed the dictates of the National Animal Welfare Law of Korea.

### Experimental procedures

**Fig. 1:**
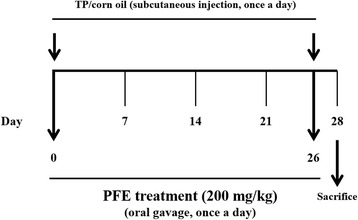
Flow diagram of the experimental procedure: a rat model of benign prostatic hyperplasia. PFE, Ponciri fructus extract; TP, testosterone propionate

The design of this experiment is outlined in Fig. 1. BPH was induced by subcutaneous injection of TP (3 mg/kg) for 4 weeks. After 1 week of acclimatization, the rats were randomly divided into four groups (*n* = 6 per group): (A) Normal control group (NC group), corn oil injection (subcutaneously, s.c.) + phosphate buffered saline (PBS) administration (peroral, p.o.); (B) control group (BPH group), TP (3 mg/kg)/corn oil injection (s.c.) + PBS administration (p.o.); (C) positive control group (Fin-treated group), TP (3 mg/kg)/corn oil injection (s.c.) + finasteride administration (10 mg/kg, p.o.); (D) experimental group (PFE-treated group), TP (3 mg/kg)/corn oil injection (s.c.) + PFE administration (200 mg/kg, p.o.). Finasteride, a 5α-reductase inhibitor and anti-BPH drug, was used as a positive control [13].

All materials were administered to rats once daily for 4 weeks, and body weight was measured weekly. The application volumes were 5 mL/kg for oral administration (PBS, finasteride and PFE) and 1 mL/kg for subcutaneous injection (corn oil and TP/corn oil) and were calculated in advance based on the most recently recorded body weights of individual rats. After the final treatment, all rats were fasted overnight and anesthetized using pentobarbital 100 mg/kg body weight injected intraperitoneally (Han Lim Pharmaceutical. Co. Ltd., Yongin, Korea). Blood samples were drawn from the caudal vena cava, and the serum was separated by centrifugation (1000 g for 15 min, 4 °C). Serum was stored at −80 °C for hormone assays. The prostates were removed immediately and weighed. Relative prostate weight was calculated as the ratio of prostate weight to body weight. The percentage inhibition of the increase in prostate weight induced by PFE was determined in accordance with a previously described method [14]. The ventral lobe of the prostate was divided in half. One half was fixed using 4% paraformaldehyde (sc-281,692; Santa Cruz Biotechnology, Santa Cruz, CA, USA) and embedded in paraffin for histomorphology and the other was stored at −80 °C for other analyses.

### Preparation of prostate homogenates

Prostatic tissue was homogenized (1/10 *w*/*v*) in tissue lysis/extraction reagent (C3228; Sigma-Aldrich) containing protease inhibitor cocktail (Ref 11 836 153 001; Roche, Mannheim, Germany) using a homogenizer (T10 Basic Ultra Turrax; IKA Works, Staufen, Germany). Homogenates were centrifuged at 15,500 g for 20 min at 4 °C. Total protein concentrations in the supernatant fractions were measured using Bradford reagent (No. 500–0006; Bio-Rad Laboratories, Inc., Hercules, CA, USA) as described previously [13].

### Elisa

The levels of testosterone and DHT in the serum or prostatic tissue were determined using an ELISA kit according to the manufacturer’s instructions. The absorbance was measured at 450 nm using a microplate ELISA reader (Bio-Rad Laboratories, Inc.). Values are expressed per mL for serum and per mg protein for the prostatic tissue.

### Histological examination

Fixed prostatic tissue embedded in paraffin wax was cut into 4 μm thick sections and stained with hematoxylin (MHS-16; Sigma-Aldrich) and eosin (HT110–1-32; Sigma-Aldrich). Coverslips were mounted on sections using mounting medium (Invitrogen, Carlsbad, CA, USA) and then the sections were examined under a microscope (Nikon, Tokyo, Japan). Prostatic epithelial thickness was measured using an image analyzer (Molecular Devices Inc., CA, USA) as described previously [13].

### Antioxidant assay

The activities of antioxidant enzymes, including CAT, GPx, GR, and SOD were quantified using commercial kits according to the manufacturer’s protocols and the results were expressed as U/mg protein as described previously [15].

### Immunoblotting

Equal amounts of total prostatic protein (30 μg) were heated at 100 °C for 5 min, loaded onto 8% sodium dodecyl sulfate–polyacrylamide gels, and electrophoresed (at 100 V for 90 min). The proteins were then transferred to an Immobilon-P polyvinylidene difluoride membrane (IPVH00010; Millipore Corporation, Bedford, MA, USA) at 25 V for 30 min using Trans-Blot Turbo Transfer System (Bio-Rad Laboratories). The membrane was blocked for 60 min with Tris-buffered saline containing 0.05% Tween-20 (TBST) plus 5% skim milk (REF 232100; BD Difco, Sparks, MD, USA), followed by incubation with anti-PCNA (1:1000 dilution), and anti-β-actin (1:1000 dilution) antibodies overnight at 4 °C. The membrane was washed three times with TBST at intervals of 10 min and then incubated with a horseradish peroxidase (HRP)-conjugated secondary antibody (PCNA, anti-mouse; and β-actin, anti-rabbit; 1:3000 dilution, respectively) (Jackson ImmunoResearch, West Grove, PA, USA) for 60 min at room temperature. The membrane was washed three times with TBST at intervals of 10 min and developed using the SuperSignal West Femto Maximum Sensitivity Substrate (ultrasensitive enhanced chemiluminescent substrate) (NCI34095KR; Thermo Fisher Scientific Inc., Waltham, MA, USA). Subsequently, membranes were photographed and, densitometric band values were determined using the commercially available ChemiDoc XRS^+^ Imaging System for quantitative analyses (Bio-Rad Laboratories) as described previously [16].

### 5α-reductase activity

In an additional experiment, a suspension of testosterone 5α-reductase was prepared from the homogenate of the ventral prostates of male SD rats, and the inhibition (%) of 5α-reductase was measured according to a method previously reported [17]. Both the Type 1 and Type 2 5α-reductase isozymes are present in the ventral prostate of rats [18]. Since the extracts of rat prostate for measurement the inhibition of 5α-reductase were used in a neutral pH buffer, 5α-reductase inhibition (%) was applied to inhibitory experiments using 5α-reductase inhibitors. Each enzyme reaction was conducted in duplicate.

### Statistical analysis

All data are presented as the mean ± standard error of the mean (SEM). Statistical significance was determined using analysis of variance (ANOVA) followed by a multiple comparison procedure and a Dunnett post hoc test. Differences in *P* values <0.05 or <0.01 were considered statistically significant [19].

## Results

### HPLC analysis of PFE for three flavonoids and three coumarins

**Fig. 2:**
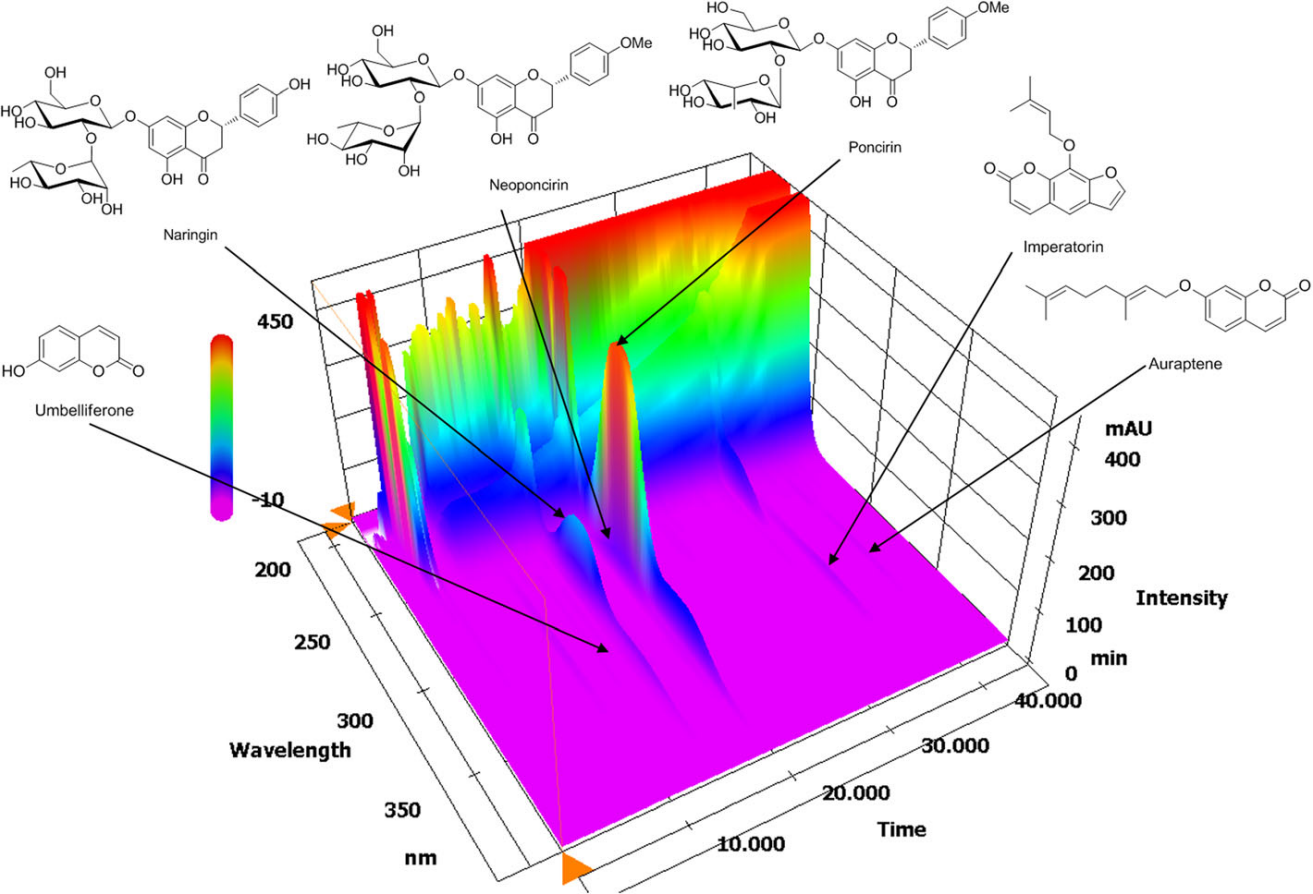
Three-dimensional chromatogram of Ponciri fructus extract by HPLC-PDA. HPLC-PDA, high-performance liquid chromatography-photodiode array detector

An established HPLC–PDA analytical method was used for the simultaneous analysis of three flavonoids (naringin, neoponcirin, and poncirin) and three coumarins (umbelliferone, imperatorin, and auraptene) in the PFE. All components were separated within 45 min using an optimized analytical method and representative three-dimensional chromatograms are shown in Fig. 2. The retention times of umbelliferone, naringin, neoponcirin, poncirin, imperatorin, and auraptene were 14.22, 14.77, 18.86, 19.37, 33.58, and 40.66 min, respectively. The correlation coefficients with the six authentic standards in the tested concentration ranges measured were ≥0.9997. Under these optimized analytical conditions, the contents of three flavonoids (naringin, neoponcirin, and poncirin) and the three coumarins (umbelliferone, imperatorin, and auraptene) in the lyophilized sample were detected at 1.17, 89.20, 28.94, 211.36, 2.25, and 15.92 mg/g, respectively.

### Effect of PFE on the prostate weight

**Fig. 3:**
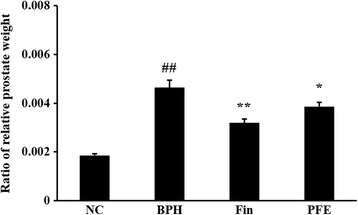
Effect of PFE on prostate weight in rats with TP-induced BPH. Ratio of relative prostate weight is the ratio of prostate weight to body weight (g/g). NC (normal control group), corn oil injection (s.c.) + PBS administration (p.o.); BPH (control group), TP (3 mg/kg)/corn oil injection (s.c.) + PBS administration (p.o.); Fin (positive control group), TP (3 mg/kg)/corn oil injection (s.c.) + finasteride administration (10 mg/kg, p.o.); PFE (PFE-treated group), TP (3 mg/kg)/corn oil injection (s.c.) + PFE administration (200 mg/kg, p.o.). Data are presented as mean ± S.E.M. (*n* = 6). Significant differences at ^##^ *P* < 0.01 compared with the NC group. Significant differences at ^*^ *P* < 0.05 and ^**^ *P* < 0.01 compared with the BPH group. BPH, benign prostatic hyperplasia; Fin, finasteride; PBS, phosphate-buffered saline; PFE, Ponciri fructus extract; p.o., peroral; s.c., subcutaneously; TP, testosterone propionate

Relative prostate weight is commonly used to evaluate the development of BPH. Rats in the TP-induced BPH group (0.0046 ± 0.0007, *P* < 0.01) showed relative prostate weights that were significantly greater than those of rats in the NC group (0.0019 ± 0.0002), whereas prostate weights of rats in the Fin-treated group (0.0032 ± 0.0004, *P* < 0.01) were decreased markedly compared with those in the BPH group. The PFE-treated group (0.0039 ± 0.0004, *P* < 0.05) also showed significant decreases in relative prostate weights compared with the BPH group. These results were similar to those for the Fin-treated group (Fig. 3).

### Effect of PFE on the levels of testosterone and DHT in serum and prostatic tissue

**Fig. 4:**
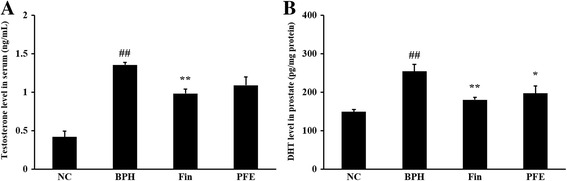
Effect of PFE on the levels of testosterone (**a**) and DHT (**b**) in serum and prostatic tissue. Individual data were obtained using an ELISA. NC (normal control group), corn oil injection (s.c.) + PBS administration (p.o.); BPH (control group), TP (3 mg/kg)/corn oil injection (s.c.) + PBS administration (p.o.); Fin (positive control group), TP (3 mg/kg)/corn oil injection (s.c.) + finasteride administration (10 mg/kg, p.o.); PFE (PFE-treated group), TP (3 mg/kg)/corn oil injection (s.c.) + PFE administration (200 mg/kg, p.o.). Data are presented as mean ± S.E.M. (*n* = 6). Significant differences at ^##^ *P* < 0.01 compared with the NC group. Significant differences at ^*^ *P* < 0.05 and ^**^ *P* < 0.01 compared with the BPH group. BPH, benign prostatic hyperplasia; DHT, dihydrotestosterone; Fin, finasteride; PBS, phosphate-buffered saline; PFE, Ponciri fructus extract; p.o., peroral; s.c., subcutaneously; TP, testosterone propionate

The major prostatic androgen is DHT, which is formed by the SRD5A2 from the influence of testosterone. As shown in Fig. 4a, rats in the TP-induced BPH group (1.36 ± 0.07 ng/mL, *P* < 0.01) showed a significant increase in serum testosterone level compared with the NC group (0.42 ± 0.17 ng/mL). By contrast, rats in the Fin-treated group (0.98 ± 0.14 ng/mL, *P* < 0.01) showed a significantly reduced serum testosterone level compared with those in the BPH group. Like rats in the Fin-treated group, those in the PFE-treated group (1.09 ± 0.24 ng/mL, not significant) showed a marked reduction in testosterone level compared with the BPH group.

The DHT level (254.56 ± 39.50 pg/mg protein, *P* < 0.01) in the prostates of rats in the TP-induced BPH group was markedly higher than in rats in the NC group (149.09 ± 12.60 pg/mg protein). However, prostatic DHT level in rats in the Fin-treated group (180.38 ± 14.81 pg/mg protein, *P* < 0.01) was significantly lower than in the BPH group. The levels of prostatic DHT in rats in the PFE-treated group (197.17 ± 43.05 pg/mg protein, *P* < 0.05) were significantly less than the levels in rats from the BPH group. These results were similar to those for the Fin-treated group (Fig. 4b).

### Effects of PFE on prostatic epithelial hyperplasia

**Fig. 5:**
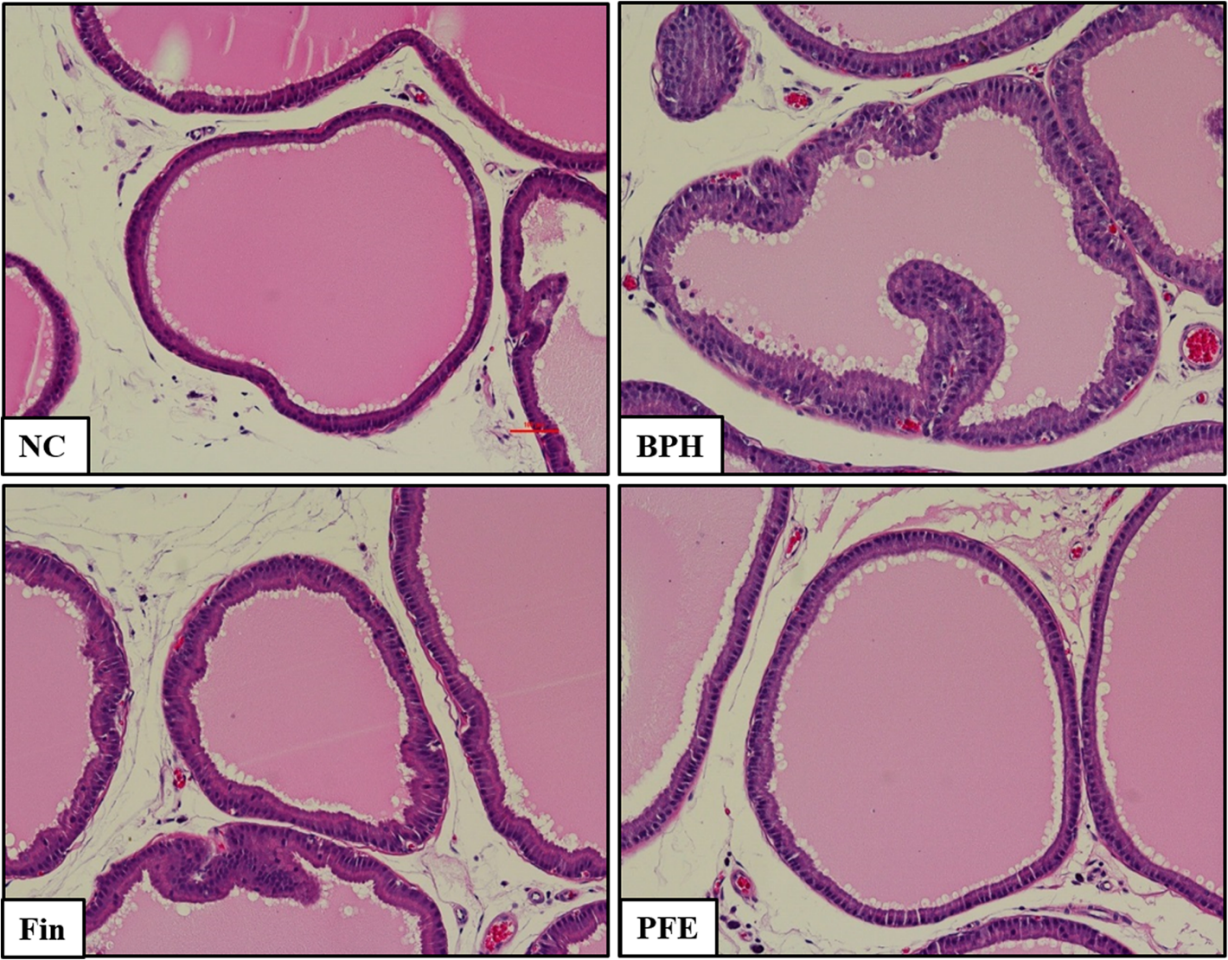
Effect of PFE on the prostatic hyperplasia in TP-induced rats. Prostatic tissues were stained with a H&E solution for histological examination (magnification, 200×). Representative photomicrographs of prostate sections are shown. NC (normal control group), corn oil injection (s.c.) + PBS administration (p.o.); BPH (control group), TP (3 mg/kg)/corn oil injection (s.c.) + PBS administration (p.o.); Fin (positive control group), TP (3 mg/kg)/corn oil injection (s.c.) + finasteride administration (10 mg/kg, p.o.); PFE (PFE-treated group), TP (3 mg/kg)/corn oil injection (s.c.) + PFE administration (200 mg/kg, p.o.). BPH, benign prostatic hyperplasia; Fin, finasteride; H&E, hematoxylin and eosin; PBS, phosphate-buffered saline; PFE, Ponciri fructus extract; p.o., peroral; s.c., subcutaneously; TP, testosterone propionate

The development of epithelial hyperplasia was evaluated by histology of prostatic lesions. As show in Fig. 5, the epithelial cell layer and lumen spaces of the prostate were larger in rats from the TP-induced BPH group compared with those from the NC group. Rats from the Fin-treated group exhibited mild epithelial hyperplasia compared with those from the BPH group. Rats from the PFE-treated group also showed a reduction in epithelial hyperplasia compared with the BPH group. Rats from the BPH group exhibited greatly increased prostatic epithelial thickness compared with those from the NC group; however, rats from the PFE-treated and the Fin-treated groups exhibited markedly reduced hyperplasia compared with those from the BPH group.

### Effect of PFE on the activities of antioxidant enzymes in prostatic tissue

**Fig. 6:**
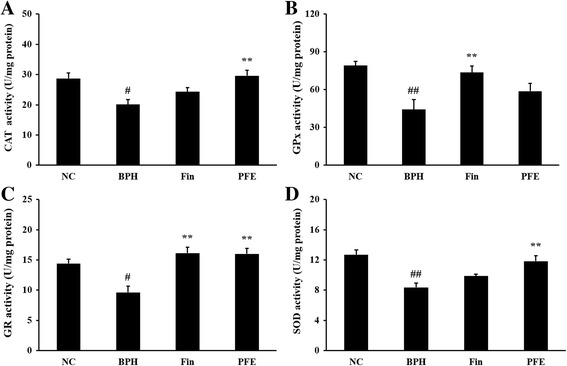
Effect of PFE on the activities of antioxidant enzymes in prostatic tissue. Individual data were obtained using assay kits: (**a**) CAT, (**b**) GPx, (**c**) GR, and (**d**) SOD. NC (normal control group), corn oil injection (s.c.) + PBS administration (p.o.); BPH (control group), TP (3 mg/kg)/corn oil injection (s.c.) + PBS administration (p.o.); Fin (positive control group), TP (3 mg/kg)/corn oil injection (s.c.) + finasteride administration (10 mg/kg, p.o.); PFE (PFE-treated group), TP (3 mg/kg)/corn oil injection (s.c.) + PFE administration (200 mg/kg, p.o.). Data are presented as mean ± S.E.M. (*n* = 6). Significant differences at ^#^ *P* < 0.05 and ^##^ *P* < 0.01 compared with the NC group. Significant differences at ^**^ *P* < 0.01 compared with the BPH group. BPH, benign prostatic hyperplasia; CAT, catalase; Fin, finasteride; GPx, glutathione peroxidase; GR, glutathione reductase; PBS, phosphate-buffered saline; PFE, Ponciri fructus extract; p.o., peroral; s.c., subcutaneously; SOD, superoxide dismutase; TP, testosterone propionate

To investigate the effects of PFE on the radical-scavenging antioxidant system in the TP-induced model of BPH, antioxidant assays were performed. The activities of antioxidant enzymes including CAT, GPx, GR, and SOD were significantly lower in prostates from rats in the TP-induced BPH group (20. 09 ± 3.72, 44.30 ± 17.41, 9.64 ± 2.29, and 8.38 ± 1.26 U/mg protein, respectively) compared with those from rats in the NC group (28.73 ± 3.98, 79.23 ± 7.60, 14.41 ± 1.78, and 12.71 ± 1.53 U/mg protein, respectively). GPx and GR activities were significantly higher in prostates from rats the Fin-treated group (73.71 ± 11.37 and 16.14 ± 2.19 U/mg protein, respectively) compared with those from rats in the BPH group. CAT, GR, and SOD activities were significantly higher in rats in the PFE-treated group (29.64 ± 4.43, 16.00 ± 2.04, and 11.82 ± 1.70 U/mg protein, respectively) compared with rats in the BPH group. GPx activity was not significantly increased in prostates from rats in the PFE-treated group (58.88 ± 13.73 U/mg protein) compared with those from rats in the BPH group (Fig. 6a–d).

### Effect of PFE on the expression of PCNA in prostatic tissue

**Fig. 7:**
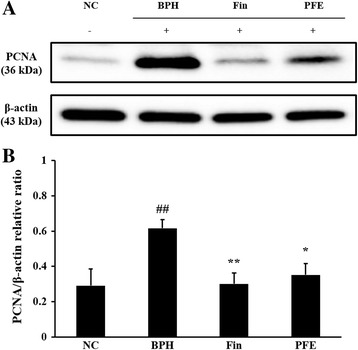
Effect of PFE on the expression of PCNA in prostatic tissue. Prostatic tissues were homogenized and PCNA/β-actin protein expression was determined using western blotting: (**a**) expressions of PCNA and β-actin, and (**b**) relative ratio, relative levels of PCNA. The β-actin protein was used as a loading control for quantitative analysis. The relative ratios of protein expression were normalized to β-actin and are represented in quantitative graphs. NC (normal control group), corn oil injection (s.c.) + PBS administration (p.o.); BPH (control group), TP (3 mg/kg)/corn oil injection (s.c.) + PBS administration (p.o.); Fin (positive control group), TP (3 mg/kg)/corn oil injection (s.c.) + finasteride administration (10 mg/kg, p.o.); PFE (PFE-treated group), TP (3 mg/kg)/corn oil injection (s.c.) + PFE administration (200 mg/kg, p.o.). Data are presented as mean ± S.E.M. (*n* = 6). Significant differences at ^##^ *P* < 0.01 compared with the NC group. Significant differences at ^*^ *P* < 0.05 and ^**^ *P* < 0.01 compared with the BPH group. BPH, benign prostatic hyperplasia; Fin, finasteride; PBS, phosphate-buffered saline; PFE, Ponciri fructus extract; p.o., peroral; s.c., subcutaneously; PCNA, proliferating cell nuclear antigen; TP, testosterone propionate

To determine the correlation between the activation of PCNA and prostatic hyperplasia in the TP-induced model of BPH, western blots were performed. As shown in Fig. 7a, the expression of PCNA protein was increased in prostates from rats in the TP-induced BPH group compared with those from rats in the NC group. Prostates from rats in the Fin-treated group had markedly reduced expression of PCNA compared with those from rats in the BPH group. Prostates from rats in the PFE-treated group also exhibited a reduction in the expression of PCNA compared with those from rats in the BPH group, similar to what was observed in prostates from rats in the Fin-treated group. The relative ratios of PCNA/β-actin were significant increased prostates from rats in the group with TP-induced BPH (0.62 ± 0.13, *P* < 0.01) compared with those from rats in the NC group (0.29 ± 0.21). By contrast, rats in the Fin- and PFE-treated groups (0.30 ± 0.13 and 0.35 ± 0.16, *P* < 0.01 and *P* < 0.05, respectively) exhibited a significantly reduced relative ratio of PCNA/β-actin compared with rats in the BPH group (Fig. 7b).

### Effects of PFE on 5α-reductase activity

To compare the inhibitory activity of PFE, Rib (nonsteroidal positive control) and Fin (steroidal positive control) against enzyme suspension of 5α-reductase prepared from prostates of rats, enzyme assay was performed. As shown in Table 1, PFE showed a weak inhibitory activity (31.8 ± 15.0%), whereas nonsteroidal (Rib, 89.1 ± 18.1%) and steroidal (Fin, 94.3 ± 46.1%) positive controls showed potent inhibition.

**Table 1:** Inhibitory effects of PFE, Rib and Fin on 5α-reductase activity in rat prostate

Group	5α-reductase inhibition (%)
PFE	31.8 ± 15.0
Rib	89.1 ± 18.1
Fin	94.3 ± 46.1

The effect of PFE (PFE-treated group) on rat prostatic 5α-reductase was tested at a concentration of 0.25 mg/mL. A nonsteroidal-positive control with Rib (riboflavin-treated group) of 1000 μM and a steroidal-positive control with Fin (finasteride-treated group) of 100 nM. Values are means of results from two different experiments

## Discussion

In the present study, we evaluated the antiproliferative and antioxidant effects of PFE on the development of BPH using a TP-induced model of BPH in rats. Rats with TP-induced BPH showed an increased relative prostate weight, elevated testosterone and DHT levels, prostatic epithelial hyperplasia, decreased the activities of antioxidant enzymes and overexpression of PCNA. However, we found that oral administration of PFE effectively prevented the progression of BPH caused by testosterone. Treatment of PFE inhibited the development of BPH, which was observed by decreased relative prostate weights, reduced testosterone and DHT levels in serum and prostatic tissue, and inhibited expression of PCNA. Histological changes also indicated that PFE treatment influenced mild prostatic epithelial hyperplasia. In addition, PFE showed mild 5α-reductase inhibitory activity.

BPH is a noncancerous and precancerous prostate condition caused by the overgrowth of prostatic epithelial and stromal cells, which results from an imbalance between proliferation and apoptosis of cells in the prostate [20]. Prostatic enlargement as increased prostate weight has been used as a vital marker of BPH progression [21]. The relative prostate weight used in the present study was the absolute prostate weight to body weight ratio. In this study, BPH induced by testosterone markedly increased in the prostate volume, which is consistent with previous studies [8, 14]. Our results demonstrated that PFE treatment significantly decreased the relative prostate weight. Histological examination of prostate tissues paralleled the results of prostate weight measurement. In support of these results, histological findings indicated that administration of PFE remarkably attenuated the prostatic epithelial hyperplasia, which reduces prostate volume or size.

Testosterone and DHT are steroid hormones related to BPH and play an important role in the development of internal male reproductive organs [4, 5]. Testosterone is converted into DHT by the 5α-reductase enzyme responsible for the development of the prostate and pathogenesis of BPH. DHT, a derivative of testosterone, stimulates cell proliferation and growth in the prostate and is a major cause of rapidly enlarged prostate. As such, DHT is considered to be the most important prostatic hormone in development and progression of BPH [22]. Additionally, steroid hormones and growth factors play a major role in regulating a variety of cellular processes that can lead to cell growth, proliferation, and differentiation [23]. Growth factor-induced PCNA is a major proliferative marker and plays a key role in certain pathological/physiological processes [7]. Consistent with previous studies [7, 13, 24], we found that administration of PFE significantly decreases the levels of steroid hormones, such as testosterone and DHT, resulting in increased 5α-reductase inhibitory activity and attenuating the expression of PCNA. Therefore, these results demonstrate that PFE should be considered as an effective antiproliferation agent in the treatment of prostatic hyperplasia.

Acute or chronic inflammation leads to proliferative events by stimulating cell growth and development through oxidative stress resulting from the overproduction of reactive oxygen species. Antioxidant enzymes, such as CAT, GPx, GR, and SOD normally protect prostatic cells, which activate the body’s defense system to damage caused by oxidative stress [25]. Antioxidant enzymes function as scavengers of free radicals by converting them to less harmful oxygen species [26]. Higher activities of antioxidant enzymes have an antioxidant effect by the alleviation of oxidative stress [7, 25]. PFE treatment significantly the increased activities of antioxidant enzymes responsible for the reduction of oxidative damage in prostatic tissue induced by testosterone. Thus, PFE could effectively reverse the changes in activities of antioxidant enzymes, which act as antioxidant agents.

Ponciri Fructus is a widely used a traditional herbal medicine. It has been reported that three flavonoids (naringin, neoponcirin and poncirin) and three coumarins (umbelliferone, imperatorin and auraptene) isolated from the 70% ethanolic extract of Ponciri fructus have various pharmacological actions including antioxidant [27], anxiolytic-like [28], anticancer [29], and anti-inflammatory effects [30–32]. Therefore, we propose that PFE can have antiproliferative and antioxidant effects based on the various pharmacological activities of the chemical-chemical interactions on the development and progression of lesions in a TP-induced model of BPH.

## Conclusions

In summary, the findings of our present study demonstrated that PFE significantly reduced the relative prostate weight, prostatic hyperplasia, level of testosterone and DHT closely related to 5α-reductase inhibitory activity, expression of PCNA, and significantly increased activities of antioxidant enzymes. These findings suggest that PFE exerts antiproliferative and antioxidant effects in preventing the development and progression of BPH.

## Abbreviations

ANOVA: analysis of variance
BPH: Benign prostatic hyperplasia
CAT: catalase
DHT: dihydrotestosterone
ELISA: enzyme-linked immunosorbent assay
Fin: Finasteride
GPx: glutathione peroxidase
GR: glutathione reductase
HPLC: High-performance liquid chromatography
p.o.: peroral
PBS: phosphate buffered saline
PCNA: proliferating cell nuclear antigen
PFE: Ponciri Fructus extract (PFE)
Rib: riboflavin
s.c.: subcutaneously
SD: Sprague Dawley
SEM: standard error of the mean
SOD: superoxide dismutase
TP: testosterone propionate
